# Biorecycling of polyethylene (PE): an integrated effort in pretreatment, degradation, and upcycling

**DOI:** 10.3389/fbioe.2025.1692651

**Published:** 2025-11-10

**Authors:** Umer Abid, Julie Gibbons, Jiansong Qin, Dongming Xie

**Affiliations:** Department of Chemical Engineering, University of Massachusetts Lowell, Lowell, MA, United States

**Keywords:** polyethylene, biodegradation, PEases, upcycling, metabolic engineering

## Abstract

Polyethylene (PE) is one of the widely utilized plastics globally, valued for its durability but unsustainable due to its resistance to biodegradation in a natural environment, leading to severe environmental accumulation. Recent studies have identified microorganisms, insects, and potential PE-degrading enzymes (PEases) capable of breaking down PE, suggesting a possible route for biorecycling. However, research in this area remains in its early stages, with limited understanding of the enzymatic mechanisms involved and the degradation products formed. A major barrier lies in the chemically inert nature of PE’s carbon–carbon and carbon–hydrogen bonds, which makes enzymatic degradation particularly challenging and unlikely to occur through a single enzyme. Overcoming these limitations requires the discovery and engineering of complex enzymatic pathways, supported by emerging tools such as omics technologies, structure-guided design, and computer-aided enzyme engineering. In parallel, the biotechnological upcycling of PE waste into value-added chemicals, by first breaking down PE into smaller products and then using them as microbial feedstocks, holds significant potential but is currently underexplored. To date, polyhydroxyalkanoate (PHA) remains the most studied PE waste upcycled biopolymer product, with only a few other studies showing production of diacids, protein, wax esters, and lipids. This highlights the need for expanded research into microbial metabolism and metabolic engineering to enable more diverse and efficient PE waste bioconversion routes. This review summarizes the current state as an integrated effort for biorecycling of PE, including PE pretreatment technologies, enzymatic PE degradation, microbial PE degradation, and PE upcycling into value-added chemicals via metabolic engineering. This review also highlights key scientific challenges and outlines future directions for PE degradation and transforming PE waste into valuable and sustainable products.

## 1 Introduction

PE and plastics in general have become one of the most widely used materials due to their low cost, high durability, and high strength. In the year 2020 alone, 400 million tons of plastic were produced on a global scale (Yao et al., 2022b). This figure accurately reflects PEs’ appearance in products used every day, like shopping bags, water and toiletry bottles, as well as foams for insulation (Elahi et al., 2021; Norton, 2021; Bernat, 2023). Most such products are intended for single use and are discarded at a high rate. On average, 12% of municipal solid waste can be attributed to plastics, the majority of which is PE (Verma et al., 2016). PE is estimated to account for about 41% of plastic waste in landfills (Wojnowska-Baryła et al., 2022). Due to the inherent non-biodegradability of PE, it must be incinerated, landfilled, or recycled using thermochemical and mechanical methods. Incinerating PE releases a surplus of toxic gases into the atmosphere, such as bisphenols, phthalates (Cosier, 2022), mercury, and polychlorinated biphenyls (Verma et al., 2016). These airborne pollutants are a threat to all life as they settle on crops and in waterways, eventually entering marine habitats and human foods (Ozdemir and Yel, 2023). Microplastics, which have been linked to landfill leachate, pollute our soil and waterways, causing great harm to surrounding ecosystems (He et al., 2019). Globally, it is estimated that each year humans consume microplastics in foods at a rate of five trillion plastic bags due to such pollution (Wojnowska-Baryła et al., 2022). To combat this, mechanical and chemical recycling methods have been largely implemented.

Current recycling methods begin with the sorting of the gathered post-consumer waste (Naderi Kalali et al., 2023). This step often introduces the most uncertainty. In 2016, it was stated that only 16% of all discarded plastic waste was successfully collected (Schyns and Shaver, 2021). Collected plastics must then be sorted and washed before being mechanically or chemically altered, requiring energy and water consumption (Altieri et al., 2021). The wastewater must also be treated. If the waste is to be mechanically altered, there is a limit to the quality/variety of potential products (Altieri et al., 2021; Ackerman and Levin, 2023). In the event of chemical recycling, additional chemicals are added, leading to more waste to be separated and disposed of (Ackerman and Levin, 2023). PE production is projected to reach 121.4 million tons by the year 2026, millions of tons of which will continue to accumulate in landfills and marine environments (Yao et al., 2022b). There are no statistical signs that suggest the production of plastics (such as PE) will slow in the projected future. For the sake of all carbon-based lives, a better, eco-friendly, and sustainable solution must be implemented to cope with the rising PE waste problem.

Biorecycling and upcycling have substantial advantages over conventional recycling methods and are part of the solution to the current plastic pollution crisis. Biorecycling starts from the use of enzymes and/or microorganisms to break down PE wastes, and the generated monomers or small molecules can be used as feedstocks for further microbial conversion into value-added products via biomanufacturing (Buragohain et al., 2020; Kawai, 2021; Gomes, 2022; Pellis et al., 2023; Patel et al., 2024). In this way, biorecycling promotes a circular economy while also providing an environmentally friendly way to lessen harmful plastics in our ecosystems (Elahi et al., 2021; Soong et al., 2022; Peng et al., 2023). PE is notoriously resistant to biodegradation due to its stable chemical structure. Pretreatment of PE has been found beneficial to help initiate the biodegradation process. Pretreatments can be achieved by chemical, physical, or biological/enzymatic means (Buragohain et al., 2020), which introduce reactive sites, reduce molecular weight, and increase hydrophilicity (Ciuffi et al., 2024). Pretreatments have proven to have a significant positive impact on PE biodegradation as well as its upcycling yield of value-added chemicals. UV pretreatment, one of the industrially applicable techniques, can alter structural, morphological, and molecular properties of PE films, as reported in the literature, resulting in 29.5% weight loss, as opposed to 15.8% without UV treatment, when exposed to mixed microorganisms (*Lysinibacillus*, *Xylanilyticus*, and *Asperillus niger*) for 126 days (Esmaeili et al., 2013). Rising concerns over the reproducibility of available studies on enzymatic and microbial PE degradation, coupled with criticisms of the analytical techniques adopted to demonstrate PE degradation, highlighted the need for more robust strategies (Jendrossek, 2024; Stepnov et al., 2024). Redirecting research efforts toward viable PE upcycling routes that produce value-added chemicals represents a promising and practical direction. Unlike traditional recycling, upcycling is a multistage process that typically involves PE breakdown into small molecules via pyrolysis or thermochemical methods and then transforming those molecules into high-value chemicals (Vanaraj et al., 2025). Depending on the microbial strain, value-added products from PE upcycling can include proteins (Byrne et al., 2022), polyhydroxyalkanoates (PHAs) (Guzik et al., 2021), waxes (Gregory et al., 2022), and long to medium chain diacids (Yeo et al., 2024). These value-added products can be used in cosmetics, biofuels, pharmaceuticals, and textiles (Huf et al., 2011; Inui et al., 2017; Sharma et al., 2021).

**FIGURE 1:**
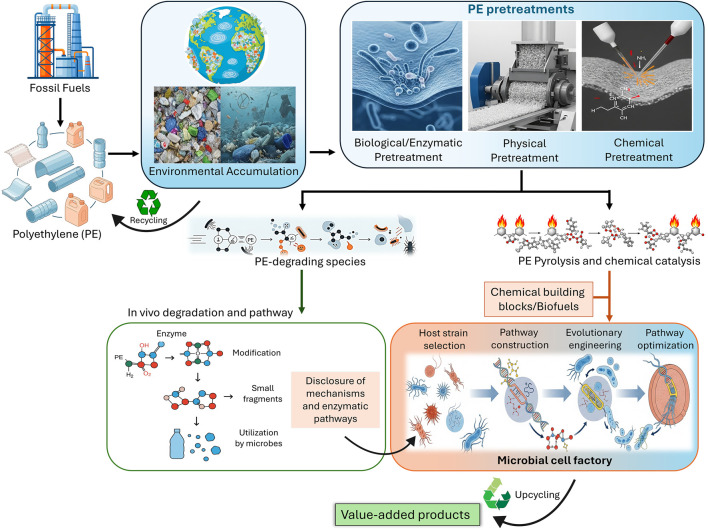
An overview of global PE flow: production, consumption, environmental accumulation, recycling, pretreatment, and upcycling.

This review aims to provide a comprehensive analysis of the PE biorecycling pipeline. The critical role of pretreatment technologies for effective biological attack, the current state of enzymatic and microbial degradation, and the specific enzymes and microorganisms that have shown promise in breaking down PE are discussed in detail. While biodegradation is critical for biorecycling, we particularly emphasize the upcycling of PE-degradation products into value-added chemicals and biomaterials using metabolically engineered strains. The entire process, from virgin PE production and its environmental accumulation to its degradation and ultimate conversion into value-added chemicals through biological routes, is conceptualized in Figure 1, which serves as a visual map for the discussion to follow. The significant challenges and future perspectives in scaling up these bioprocesses have also been elaborated.

## 2 Pretreatment technologies for enhanced biodegradation

PE is a widely utilized, unreactive plastic material due to its high durability and great resistance. This high resistance of PE is owing to the exclusive C-C linear carbon atoms backbone and C-H covalent bonds that are highly stable (Ellis et al., 2021). This unique property of PE makes them highly durable during their use phase; however, it causes challenges in their recycling and upcycling (Lemmens et al., 2024). Furthermore, the molecular weight of PE, like high-density polyethylene (HDPE) with high molecular weight, poses a significant recycling and upcycling challenge by making it more complicated for oxidizing agents, enzymes, and microorganisms to access long and dense polymer chains (Sudhakar et al., 2008; Fontanella et al., 2010). Additionally, PE resistance to biodegradation is attributed to the tightly packed crystalline regions due to a high degree of crystallinity along with a low available specific surface area and high hydrophobicity (Burelo et al., 2023). These physical characteristics of PE can be modified using physicochemical and microbial pretreatment processes or a hybrid of both. Physicochemical pretreatment involves the addition of an oxidizing agent, hydrolysis reactions, UV exposure, and thermal treatment. These pretreatments alter the PE structure, surface features, and crystallinity, resulting in improved PE hydrophilicity, affinity to microbes, and reactive surface sites, therefore, leading to high PE biodegradation and upcycling (Restrepo-Flórez et al., 2014; Bardají et al., 2020; Burelo et al., 2023).

### 2.1 Physical pretreatment methods

#### 2.1.1 UV radiation (photo-oxidation)

Pretreatment using ultraviolet (UV) radiation is considered one of the most environmentally friendly pretreatment techniques. The exposure of PE to UV results in the generation of oxygen-free radicals within the PE chains, which can take part in the polymeric chain reaction pathways (initiation, propagation, and termination) to result in the formation of comparatively shorter chain molecules, including olefin and ketones (Vasile and Pascu, 2005; Gewert et al., 2015; Erdmann et al., 2020). These small molecules, as a result of photo-oxidation, are then more easily accessible to be attacked by microbial enzymes for further consumption and degradation (Taghavi et al., 2021b). Furthermore, higher microbial colonization and improved hydrophilicity of PE were also observed by UV radiation pretreatment (Suresh et al., 2011; Ciuffi et al., 2024). The UV oxidation mechanism of plastic has been well-studied in past studies (Gewert et al., 2015; Taghavi et al., 2021a; Taghavi et al., 2021b). However, several studies also reported sunlight as a UV pretreatment and degradation method (Abd El-Rehim et al., 2004; Gong et al., 2019). Comparative studies conducted by Doğan (2021) demonstrated higher degradation of PE using artificial UV light in a shorter time compared to sunlight. Even with a general agreement on weathering and PE oxidation under sunlight, the low-energy UV from the sun is not as effective as the higher-energy UV from the artificial UV radiation sources (Jones, 2002; Doğan, 2021). UV treatment of LDPE sheets for 2.1 days with 354 nm UV resulted in the improvement of their degradation (weight loss) from 9% to 44% in 90 days by the fungi *Curvularia lunata* SG1 (Raut et al., 2015). An increase from 15.8% to 29.5% in the biodegradation of LDPE films was reported by mixed bacteria in 126 days after pretreatment of LDPE films using 55 W UV lamps irradiation for 25 days (Esmaeili et al., 2013). For the upcycling of PE into value-added products, the study revealed that the UV light treatment of LDPE powder enhanced its bioconversion by *Cupriavidus necator* H16, which was able to accumulate poly(3-hydroxybutyrate-co-3-hydroxyvalerate) (PHB-V) up to 3.18% ± 0.4% of cell dry mass (Montazer et al., 2019). Table 1 shows several PE microbial degradation studies using UV-pretreated PE, summarizing PE weight loss (%) performance with several microbial strains under different PE UV-pretreatment conditions.

**TABLE 1:** Major PE pretreatments and their effects on PE biodegradation.

Pretreatment technology	Sample type	Degradation approach	Pretreatment conditions	Degradation time	Wt. loss (%) - pretreated	Wt. loss (%) - unpretreated	Ref.
Ozonation	LDPE films	Fungi *Aspergillus* sp.	Gamma rays 1,000 kGy	90 days	∼3.6%	None	Sheik et al. (2015)
		Fungi *Paecilomyces lilacinus*			∼2.0%	None	
		Fungi *Lasiodiplodia theobromae*			∼3.0%	None	
Physico-chemical (oxidation)	LDPE pellets	Yeast *Y. lipolytica* DSM 8218	1%–10% anionic surfactant (60 days)	30 days	0.20%	0.12%	Buron-Moles et al. (2025)
		Yeast *Y. lipolytica* DSM 3286			0.20%	0.16%	
UV	PE shopping bag	Microbial consortium	245 nm UV (72 h, 24 cm sample distance)	90 days	∼6.5%	Not discussed	Taghavi et al. (2021b)
			245 nm UV (72 h, 12 cm sample distance)		∼7%		
			245 nm UV (120 h, 12 cm sample distance)		∼8.5%		
UV	LDPE films	Fungi *Penicillium* sp., *Rhizopus arrhizus*	<300 nm UV (10 days)	180 days	∼3.5%	∼1.7%	Mahalakshmi and Andrew (2012)
UV	LDPE films	Fungi *Curvularia lunata* SG1	354 nm UV (2.1 days)	90 days	∼44%	∼9%	Raut et al. (2015)
Photocatalytic (TiO_2_ and 5M NaOH)	LDPE powder	UV irradiation	254 nm, 5 mW cm^−2^ UV	800 h	∼87%	∼55%	Lu et al. (2025)
UV	LDPE film	Bacterial consortia PB1, PB2, PB3, LS	UV-C irradiation (15 W, 50 Hz), 40 h	120 days	2.2%–5.27%	1.3%–2.8%	Muangchinda and Pinyakong (2024)
Sunlight			Sun exposure of 40 h		1.7%–4.6%		
Thermal			60 °C (hot air-oven), 40 h		1.4%–3.2%		
UV	LDPE film	Mixed Bacteria *Lysinibacillus xylanilyticus* and *Aspergillus niger*	Two 55 W UV lamps irradiation, 25 days	126 days	29.5%	15.8%	Esmaeili et al. (2013)
Ozonation/O_2_-oxidation	LDPE film	Bacteria *Pseudomonas* sp. Rh926 (strain 15G3)	80 °C for 20 h, flow rate of 0.4 L/min	30 days	25%	3%	Jha et al. (2024)
UV with acid	PE	Enzymes laccase and manganese peroxidase from *P. simplicissimum*	Not discussed	90 days	38%	Not discussed	Sowmya et al. (2015)
Autoclaving			Not discussed		16%		
Surface-sterilization			Wash with ethanol for 1 min and sodium hypochlorite solution for 3 min		7.7%		
Thermal	LDPE film	Bacteria *B. sphericus*	80 °C for 10 days	12 months	∼19%	∼10%	Sudhakar et al. (2008)
		Microorganism (*B. cereus*)			∼15%	∼5%	
	HDPE film	Bacteria *B. sphericus*		5 months	∼9.6%	∼3.5%	
		Bacteria *B. cereus*		12 months	∼7%	∼2.1	

#### 2.1.2 Thermal treatment

Thermal treatment is another viable way of boosting PE degradation as it modifies the properties such as morphology and crystallinity, and creates oxidized groups on the PE surface. As described in several studies, partial degradation of PE can be accomplished after thermal pretreatment (Albertsson et al., 1998; Awasthi et al., 2017a). Thermal pretreatment of PE is usually carried out in a hot air oven operated around 60 °C–150 °C for a long time, similar to UV pretreatment (Awasthi et al., 2017b; Crystal Thew et al., 2024; Muangchinda and Pinyakong, 2024). Oxidized groups, including hydroxyl, carboxyl, and carbonyl groups, are formed along with the reduction of PE chain size as a result of thermal treatments. Microorganisms can then attack these oxidized groups and degrade PE more effectively (Albertsson et al., 1995). The microbial and enzymatic attachment to PE is modulated by the presence of oxidized groups, which reduce the overall PE surface hydrophobicity (Tribedi and Sil, 2013; Awasthi et al., 2017a). The incubation of thermally pretreated HDPE with *B. sphericus*, showed an increase in the degradation, from 3.5% (untreated) to 9%, when incubated (Sudhakar et al., 2008). The 60-day incubation of HDPE films with *K. pneumoniae* CH001 manifested thick biofilm formation on the HDPE surface and 18.6% degradation after thermal pretreatment of 10 days in a hot air oven at 70 °C, oxidizing the HDPE chains (Awasthi et al., 2017b). The formation of biofilm increases the surface hydrophilicity of PE, resulting in a faster degradation rate. Another study reported that increasing the pretreatment temperature caused more fungal filaments (hyphae) to grow on the surface of the low-density polyethylene (LDPE) (Manzur et al., 2004). In comparison to UV pretreated LDPE, a study found a higher number of hydroxyl and carbonyl groups contained by thermally pretreated LDPE (Manzur et al., 2004). Thermally oxidized PE wax supplemented with tryptone soya broth (TSB) resulted in the production of 1.24 g/L PHA by *C. necator* H16 compared to 0.39 g/L PHA without thermally oxidized PE wax (Radecka et al., 2016). A study conducted by Torres-Zapata et al. (Torres-Zapata et al., 2022) demonstrated the conversion of PE to triglycerides (TGs) through a thermochemical and biotechnological procedure where hydrothermally processed PE oil was utilized as a carbon source by *Yarrowia lipolytica* to improve biomass growth and TGs production yield by 130%. Furthermore, several PE microbial degradation studies using thermal pretreated PE are also illustrated in Table 1, summarizing PE weight loss (%) performance with several microbial strains under different thermal pretreatment conditions.

### 2.2 Chemical pretreatment methods

#### 2.2.1 Acid/alkaline treatment

Chemical pretreatment of PE is accomplished by exposing PE to highly concentrated acids and solutions that induce PE oxidation by acting as an oxidizing agent. Chemical pretreatments can create polar and unsaturated groups on the surface of PE (Crystal Thew et al., 2024). The use of strong acids results in the liquid etching of the PE surface, thus enhancing the formation of surface cracks/pits and overall surface roughness (Mijovic and Koutsky, 1977). Although reports mention significant PE surface etching using strong acids at higher temperatures of above 60 °C, chemical pretreatments are preferred at lower temperatures (Mijovic and Koutsky, 1977; Balasubramanian et al., 2014). Several studies have reported the use of chemical pretreatment as part of a cascade PE pretreatment, rather than a sole pretreatment method. A study carried out by Balasubramanian et al. (2014) reported the use of cascade pretreatment for HDPE films by subjecting films to UV for 60 h, followed by 50 °C for 70 h thermal pretreatment, and chemical pretreatment using KMnO_4_/HCl and citric acid. Nitric acid (99.0%) at 80 °C was utilized by Hasan et al. (2007) for cascade pretreatment of UV-treated LDPE. Pretreated LDPE was utilized as the sole carbon source for growth and was inoculated in a medium containing *Fusarium* sp. AF4. The fungus growth and structural properties were analyzed. When the fungus *P. citrinum* was used on the LDPE pretreated with nitric acid, the biodegradation was significantly more effective, with a weight loss of around 47.22% compared to 38.82% without pretreatment (Khan et al., 2023).

### 2.3 Alternative pretreatment methods

In addition to the adopted and well-explored conventional pretreatment methods, several emerging methods can also improve the overall degradation efficiency of PE. The use of ionizing radiation (gamma-ray) is reported to be an effective method for the formation of free radicals, reactive intermediates, and excited states in plastic (Crystal Thew et al., 2024). The species generated owing to the excitation of the plastic modifies the structure by undergoing a crosslinking or chain scissoring mechanism. Exposure of LDPE to 1000 kGy gamma radiation induced the generation of a carbonyl group within LDPE, which helped enhance the biodegradation when incubated with *Lasiodiplodia theobromae* (Sheik et al., 2015). Surface plasma treatment is another emerging technique to improve surface roughness, radical formation, and increase the hydrophobicity of the plastic (Abourayana and Dowling, 2015). Thermo-irradiation pretreatment, involving the exposure of LDPE and LLDPE to high-energy gamma rays followed by their thermal treatment at 150 °C and 90 °C for 7 days, was carried out for the enhancement of biodegradation of LDPE and LLDPE using *Bacillus amyloliquefaciens* (Novotný et al., 2018). A decrease in the LLDPE dry weight by 1.1 ± 0.3 to 3.2 ± 1.3% was observed within 40–60 days of inoculation, along with the appearance of carbonyl groups in the FTIR spectra. Biodegradation of plasma-pretreated LDPE sheets was investigated using *Pleurotus ostreatus* (Gómez-Méndez et al., 2018). Oxygen glow plasma was utilized, at 600 V for exposure of 6 min, for the LDPE sheets pretreatment. A 76.57% decrease in the surface contact angle of the LDPE sheet was observed after plasma pretreatment due to the improved LDPE surface hydrophobicity. *Pleurotus ostreatus* colonization was increased from 45.55% to 88.72% after plasma pretreatment (Gómez-Méndez et al., 2018). Surface roughness was also boosted by 99.81% after pretreatment. These studies highlight the importance of shorter pretreatment times for plasma and gamma radiation pretreatments compared to conventional UV and thermal pretreatments, which can last several days (Table 1). Oxidative surface treatment of PE using air or oxygen has been reported to enhance the downstream production of value-added products such as PHA via upcycling. Introducing small amounts of air or oxygen during PE pyrolysis generates carbonyl and hydroxyl groups in the resulting hydrocarbons, making them more accessible for utilization by *Cupriavidus necator* H16 and potentially resulting in higher PHA titers of 1.26 g/L compared to 0.46 g/L from untreated PE waste (Hu et al., 2024).

## 3 Enzymatic degradation of PE

### 3.1 Challenges in direct enzymatic attack on native PE

**FIGURE 2:**
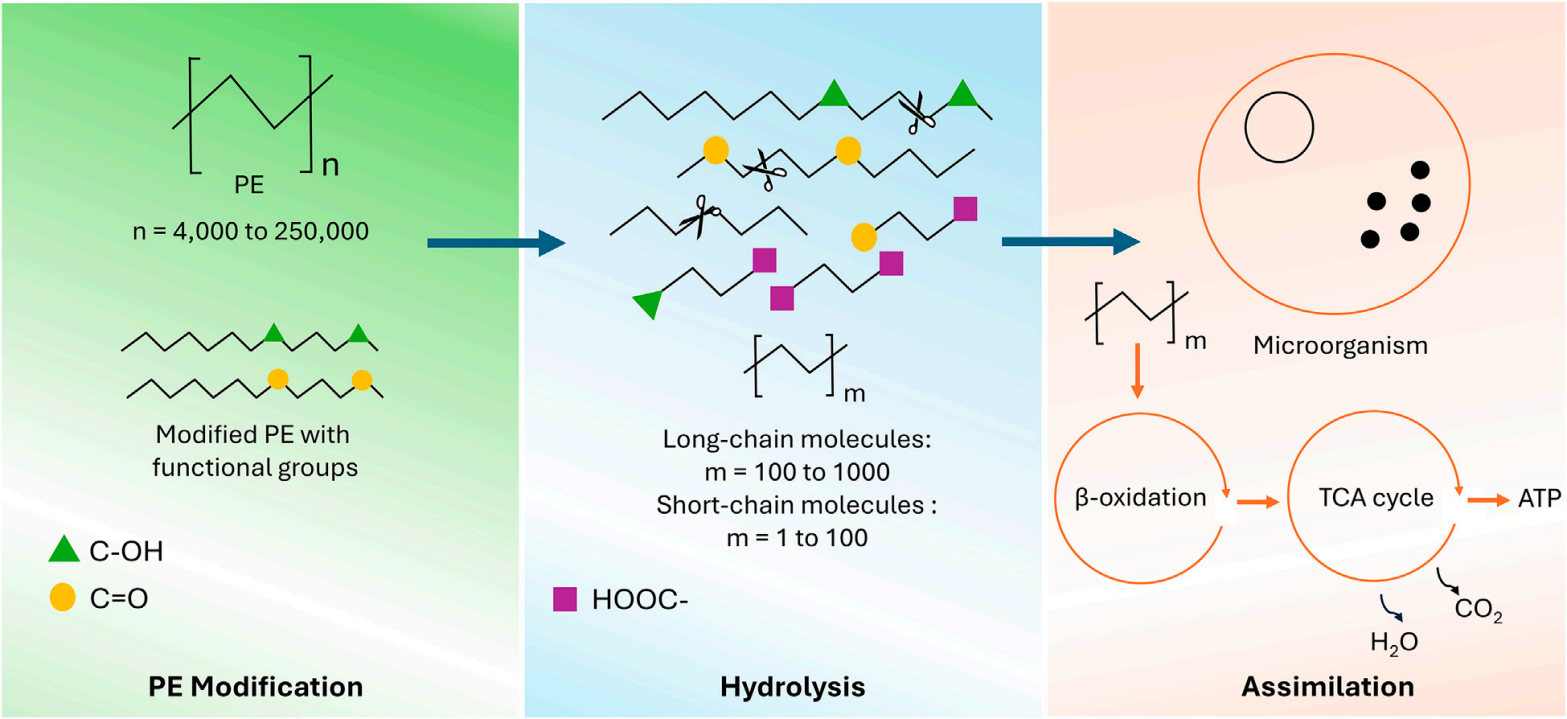
An overview of the mechanism of PE enzymatic degradation and its further assimilation.

The use of enzymes (PEase) for PE degradation is a well-known and established method for effective PE degradation. However, the availability of the research data for direct enzymatic degradation of native PE is very limited. One recent study reported the use of three enzymes from a bacterial consortium and showed the successful start of untreated PE hydrolysis (Gao and Sun, 2021). The initial step of oxidizing PE is the most difficult part of the enzymatic PE degradation process, which is attributed to the strong C-C and C-H chemical bonds. Therefore, most of the enzymatic PE degradation studies have reported the use of pretreatment technologies for oxidation initiation and to enable enzymatic degradation (Ghatge et al., 2020). This assertion needs further exploration, particularly knowing that PE does not contain any hydrolysable bonds. Hydrolases likely only take part in PE biodegradation steps that incorporate a bond that they are capable of cleaving after the pretreatments (Jin et al., 2023). Furthermore, the genetic enhancement of existing PEases and the discovery of new variants for effective PE recycling remain a big challenge with very little exploration. This is largely due to the intricate, multi-step nature of PE degradation, reliance on imprecise degradation quantification techniques, and the limited understanding of each degradation stage, which hinders the development of highly efficient enzymes (Jin et al., 2023). Figure 2 shows the overall mechanism of PE enzymatic degradation, including biodeterioration of modified PE with functional groups, depolymerization into smaller molecules, and absorption of small molecules into microorganisms.

### 3.2 Key enzyme classes involved in PE degradation

#### 3.2.1 Oxidoreductases (laccases, peroxidases, monooxygenases)

The enzymatic degradation of PE proceeds in two distinct stages: the initial adsorption of enzymes onto the PE surface, followed by the subsequent hydro-peroxidation or hydrolysis of chemical bonds (Mohanan et al., 2020). The biological sources of these PE-degrading enzymes are diverse, including microorganisms from various environments and the digestive systems of certain invertebrates (Carr et al., 2020). Oxidoreductases, a class of PEase enzymes like laccases, peroxidases, and certain monooxygenases, are promising for the successful oxidation and degradation of PE. Laccases and peroxidases, including lignin and manganese peroxidases, have been reported to play a crucial role in the oxidation of PE waste collected from the environment (Jin et al., 2023). Laccases are the most extensively studied PEases. However, more effort has been made towards the biological sources for laccase isolation compared to recombinant enzymes (Gao et al., 2022; Yao et al., 2022b; Zhang et al., 2022). Oberbeckmann et al. (2021) combined a protein analysis and genetic engineering to observe the production of laccases and manganese peroxidases during the degradation of PE. Gene sequencing for algae-bacteria (*Jacksonvillea* sp.) was carried out by Mishra et al. (2021) to find genes related to forming a biofilm and breaking down PE. This work suggested the production of a variety of enzymes, including laccases, esterases, and peroxidases, which are key to degrading PE. Degradation of PE using *R. ruber* C208 reported the exclusive presence of multicopper oxidase (laccase) in the extracellular portion, indicating laccase’s crucial role in the oxidation and degradation of PE (Santo et al., 2013). The synergistic effect of a dual enzyme system consisting of glutathione peroxidase and laccase (from a marine fungus) was studied on PE film (Gao et al., 2022). The effect of laccase from *Psychrobacter sp.* NJ228 on unpretreated PE was studied by Zhang et al. (2022). Through various analytical methods, their findings demonstrated a reduction in PE crystallinity and hydrophobicity, 13.2% weight loss in PE particles, alteration in PE morphological features, and formation of oxygen-based functional groups. The concerns with such reported PE degradation efforts using enzymes are diverse, owing to the aspects involved, such as the PE composition (e.g., portion of weight loss from degradation of oligomers and additives, instead of PE itself), as well as the limited quantity of PE material used and the swift loss of low molecular weight fractions within the PE (Liu et al., 2025).

Alkane monooxygenase (AlkB) of *Pseudomonas sp.* E4 is reported to be involved in the initial oxidation of PE by mineralization of 19.3% of the carbon PE into CO_2_ (Yoon et al., 2012). The formation of C-O and -OH functional groups on the LDPE surface after surface treatment with phenylalanine monooxygenase ensures its potential to oxidize PE, as illustrated in Figure 2. Reduction in LDPE molecular weight in *Pseudomonas aeruginosa* was also observed while treating LDPE with isocitrate lyase in combination with phenylalanine monooxygenase. Microorganisms that break down short-chain alkanes typically use methane monooxygenase (Pinto et al., 2022). For medium-chain alkanes (C_5_–C_17_), initial hydroxylation is usually catalyzed by two types of enzymes: soluble cytochrome P450s, specifically cytochrome P450 CYP153 alkane hydroxylase, and integral membrane nonheme iron monooxygenases, such as AlkB (Schneiker et al., 2006; Pinto et al., 2022). Various pathways can initiate the breakdown of PE using these PEases. These pathways include the addition of a hydroxyl group (−OH) at either end of the polymer chain (terminal or sub-terminal hydroxylation) or along the chain itself (in-chain hydroxylation). This process introduces functional groups such as alcohols, aldehydes, ketones, and carboxylic acids, which make the PE more susceptible to further degradation (Temporiti et al., 2022). Despite extensive research focusing on this class of PEases, there still remains a deficiency of thorough experimental evidence indicating the role of oxygenases in PE degradation. Moreover, the contribution of other specific enzymes in the PE degradation process has yet to be clearly established, as highlighted in a recent expert review by Jendrossek (Jendrossek, 2024).

#### 3.2.2 Hydrolytic enzymes (e.g., esterases, lipases)

Another class of PEase, known as hydrolytic enzymes, is well studied for its prominent effect on PE degradation. The participation of hydrolytic enzymes, like esterases, in the PE degradation was reported to occur after conversion of oxidized PE to an ester via Baeyer–Villiger monooxygenases (BVMO), as revealed by a PE degradation mechanism study (Zadjelovic et al., 2022). Studies reported an increase in the presence of enzymes like lipase, esterase, and cutinase during PE degradation (Jin and Jia, 2024). Esters were also found among the PE breakdown products, which suggests that these enzymes might play a role in the degradation process (Jin and Jia, 2024). However, the exact way these hydrolases help degrade PE is still a mystery. The production of bacterial esterase from three different marine bacterial isolates (*Bacillus subtilis* H-248, *Marinobacter* sp. H-244, and *Marinobacter* sp. H-246) was studied for their degradation effect on PE films. Within 90 days, the highest PE film weight loss was observed using H-246 isolate. Lipases work by the degradation of long carbon chains within the PE material. 30 kDa lipase produced from *Aspergillus niger* MG654699.1, with a biocatalytic activity of 176.55 U/mL, resulted in 3.8% weight loss of PE as described by Safdar et al. (2024). Surface deterioration, alteration in functional groups, and the physical impact of lipase were studied. Lipase, like *Pelosinus fermentans* lipase 1 (PFL1), reported by Kim et al. (2024), degraded oxidized LDPE films by cleaving the newly formed ester bonds, resulting in a reduction of weight average by 44.6% and a reduction in number average molecular weights of oxidized LDPE films by 11.3%. These hydrolytic enzymes take part in the initial and subsequent degradation stages of PE by targeting ester linkages, followed by hydrolysis and chain scission (Sutkar and Dhulap, 2025), as shown in Figure 2. Although studies have reported the activity of oxidative enzymes on PE, the idea that PE can undergo efficient enzymatic degradation remains controversial (Stepnov et al., 2024). This skepticism largely originates from the lack of independent studies that have successfully reproduced earlier findings to validate the idea.

### 3.3 Strategies for enhancing enzyme activity and stability

Metal ions play a very crucial role in enzyme-catalyzed degradation reactions, often performing as a cofactor and influencing the catalytic activity of enzymes. For instance, multi-copper oxidoreductases manifested an increase in the PEase activity with the addition of copper ions (Santo et al., 2013). Significant acceleration in PE degradation using MnP was observed with the addition of Mn(II) into the culture medium with *T. versicolor* and *P. chrysosporium* (Iiyoshi et al., 1998). Therefore, selecting the correct auxiliary agents is crucial for enhancing PEase activity. Several environmental factors, including pH value, light exposure, temperature, and oxygen, can significantly influence the degradation and enzyme activity, as these factors not only influence the enzyme performance and stability but also weaken the PE structures, making it more vulnerable to enzyme attack (Pospíšil et al., 2006; Choi et al., 2024). Directed evolution, rational, and semi-rational approaches have been discussed in the literature to potentially engineer redox potential, pH performance, and thermal stability of PEases like laccases (Mate and Alcalde, 2015; Pardo and Camarero, 2015). Aniline in laccase has also been rationally designed by computer-aided laccase engineering for high stability and to confer affinity (Santiago et al., 2016). Researchers improved the thermal stability of an evolved high-redox potential laccase from fungal sources by first replacing its second cupredoxin domain with the corresponding domain from a different fungal laccase, followed by using computational methods to design recombinant chimeras that stabilized the enzyme’s flexible surface loops (Vicente et al., 2020). Protein engineering studies for enhanced PE degradation have, unfortunately, not been documented in the literature yet, even for promising PEases like laccases. This protein engineering field represents a significant area for future research to advance the activity and stability of PEases. Furthermore, the limited understanding of the PE degradation pathways and of each step involved in it hampers the development of effective future strategies for enzyme activity improvement and the development of precise analytical techniques for the demonstration of PE degradation.

## 4 Microbial degradation of PE

### 4.1 Diversity of PE-degrading microorganisms

#### 4.1.1 Bacterial degraders *(Pseudomonas*, *Rhodococcus, and Bacillus*)

A wide range of bacteria capable of PE degradation have been isolated from landfills, compost, and marine habitats in recent years. Bacteria can degrade PE by adhering to its surface (using the substrate PE as a food source) and secreting enzymes that degrade PE and catalyze a chain of redox reactions (Hou et al., 2022; Srikanth et al., 2022). Such PE degradation enzymes include monooxygenase, hydroxylase, and dioxygenase (Hou et al., 2022). Currently, the most researched bacteria capable of PE degradation include *Pseudomonas*, *Rhodococcus*, and *Bacillus*. The *Pseudomonas* sp. AKS2 strain is reported to degrade 5% ± 1% of PE feedstock in 45 days without prior oxidation (Lv et al., 2024). However, it is noteworthy that by introducing modulating agents like mineral oil, the hydrophobic interactions can increase, causing more frequent plastic and bacterial attachment, leading to higher PE degradation (Tribedi and Sil, 2013). The same phenomenon is seen when PE is exposed to *Rhodococcus ruber*. When incubated in liquid culture for 30 days, 8% weight reduction of the PE is observed, and increased by 50% when exposed to mineral oil (Gilan et al., 2004). When LDPE was exposed to *Bacillus subtilis* ATCC6051 and *Bacillus licheniformis* ATCC14580 for 30 days, Yao et al. (2022a) reported a weight reduction of 3.49% and 2.83%, respectively. While significant development has been made, further research is required to completely comprehend the mechanisms of PE degradation and to improve the efficiency of bacterial degradation. Moreover, most of the PE degradation studies using microorganisms primarily rely on PE weight loss as an indicator of degradation (Gilan et al., 2004; Ghatge et al., 2020; Yao et al., 2022a). However, this approach is subject to criticism as the reported weight loss may potentially result from the breakdown of additives and leachate, which can constitute a significant portion of PE (Ghatge et al., 2020). Such studies should be further validated using advanced biochemical, physicochemical, and molecular biology techniques to ensure accurate assessment of true PE degradation (Weber et al., 2017; Danso et al., 2019).

#### 4.1.2 Fungal degraders (*Penicillium*, *Asperillus,* and *Phanerochaete*)

Fungi can also degrade PE in the presence of pro-oxidant ions by adhering to the PE’s surface and secreting PE-degrading and lignocellulolytic enzymes (i.e., laccase, cutinase, LiP, and MnP) (Cowan et al., 2022; Srikanth et al., 2022). Lignocellulolytic enzymes are ordinarily used to break down lignin, a complex polymer in plant cells. These enzymes have been found to degrade PE, along with a complex polymer (Temporiti et al., 2022). Similar to the degradation of PE by bacteria, the fungal degradation mechanism promotes oxidation reactions, which increase the hydrophilicity of the PE, promoting greater adhesion by the fungi (Srikanth et al., 2022). Fungi use an elongating cell structure called a hypha, which utilizes polarized exocytosis to create new cell material, to integrate with the PE surface (Steinberg et al., 2017). Fungal species that show effective degradation of PE include *Penicillium*, *Aspergillus*, and *Phanerochaete* (Srikanth et al., 2022). Sowmya et al. (2015) reported that UV-pretreated PE exposed to *Penicillium simplicissimum* showed 38% weight loss. Balasubramanian et al. (2014) addressed the use of *Aspergillus terreus* MF12 for microbial treatment of HDPE in combination with physical and chemical pretreatments. HDPE degradation of up to 20.8 ± 0.1% was observed with combined pretreatment. Bautista-Zamudio et al. (2023) reported 70% weight loss of pre-treated PE when exposed to *Phanerochaete chrysisporium* for 15 days at 37 °C. These microorganisms are capable of PE degradation by utilizing innate biological processes. Although these studies utilizing fungal degraders have demonstrated high PE weight loss through microbial activity, there remains insufficient evidence to validate whether such weight loss reflects only PE biodegradation or if it is partially attributable to the breakdown of additives and other components within the PE. This skepticism is mainly due to the lack of independent studies confirming earlier findings.

#### 4.1.3 Insect gut microbiota

Yeast and bacteria extracted from the gut of waxworms and wood-feeding termites have been observed to be capable of breaking down PE. *Enterobacter* spp., found in the wood-feeding termite gut microbiome, caused up to 81% weight loss of exposed LDPE after 120 days (Ali et al., 2024). A recent study demonstrated that dye-decolorizing peroxidases (DyPs) from the gut microbiota of mealworms were capable of initiating LDPE oxidation when fed with LDPE (Klauer et al., 2025). The gut microbiota of mealworms was found to degrade PE, with alkene groups detected in the fecal matter (frass) (Brandon et al., 2018). While the formation of these alkenes has been linked to the catalytic activity of CYP152 peroxygenases and the decarboxylase OleT, the complete mechanism of how these enzymes contribute to the overall PE degradation process remains unclear (Jin et al., 2023). Recently, it was reported that the larvae of *Achroia grisella* and *Plodia interpunctella,* and the beetle *Uloma* can degrade PE due to their ability to metabolize long-chain hydrocarbons (Yang et al., 2015; Kundungal et al., 2025). It was also reported that the wax worm *Galleria mellonella*, was capable of oxidizing and depolymerizing PE by secreting saliva containing the key PEases (Sanluis-Verdes et al., 2022). However, a revisit to this study showed that the previously observed results were misinterpreted, and whether key PEase enzymes exist in the wax worm saliva remains a question (Stepnov et al., 2024). Additionally, a potential limitation in the available body of research lies in the way PE degradation is assessed or monitored in specific and controlled laboratory conditions. For instance, studies reporting PE degradation involving *Tenebrio molitor* have demonstrated degradation in terms of PE consumption in its diet (Jin et al., 2023). Studies using insects for PE degradation utilize the persistent weight of *G. mellonella* as evidence of PE uptake by the insect for their energy requirements (Kong et al., 2019). The presence of alkene groups in the frass of PE-fed mealworms has been reported as evidence of PE degradation by the gut microbiota of mealworms (Brandon et al., 2018), instead of providing an experimental investigation more aligned towards a complete picture of PE degradation. Comprehensive experimental investigations, beyond weight measurements, are essential to fully validate the utilization of PE as an energy source by insects. Such reports thus lack a complete understanding of the PE degradation mechanism.

### 4.2 Mechanisms of microbial PE biodegradation

The general steps used by microorganisms to degrade PE are microbial adhesion, enzyme secretion, and intracellular metabolism. For microbial adhesion to occur, the hydrophobic surface of PE must be pretreated, often by UV exposure. Such pretreatment decreases the hydrophobicity, allowing the microorganisms to interact with the PE surface (Temporiti et al., 2022). Bacteria create a biofilm, a layer of cells that embed into the PE surface with the purpose of gene transcription and cell growth (Arampatzi et al., 2011). The presence of a biofilm allows the concentration of PE-degrading enzymes to localize on the PE surface, as the biofilm is also hydrophobic (Cai et al., 2023). Microbes secrete enzymes outside the cell that are capable of PE breakdown. Fungi secrete lignocellulolytic enzymes (laccases and peroxidases), which would ordinarily be used to break down lignin (Temporiti et al., 2022). Bacteria secrete oxidoreductases and hydrolases, which promote a chain of redox reactions (Hou et al., 2022; Srikanth et al., 2022). These secreted enzymes initiate the depolymerization of the PE. Microbes can also utilize degraded plastics as an energy source. The secreted exoenzymes break down the substrate (PE) into small, water-soluble molecules (monomers/oligomers) that can pass through the microbes’ cell membrane (Hou et al., 2022). Intracellularly, the small monomers are oxidized and utilized as a carbon/energy source in central metabolic pathways (β-oxidation/Krebs cycle) (Elahi et al., 2021).

### 4.3 Optimization of microbial degradation conditions

To maximize degradation, further optimization of conditions is required to increase the key enzymes’ efficiency. Raut et al. (2015) reported that optimal bioreactor conditions for microbial PE degradation should include pH 7.6, temperature of 38 °C, agitation of 190 rpm, and an incubation period of 262 days. Under such conditions, 48% degradation of LDPE by *C. lunata* SG1 was achieved. However, optimal conditions largely depend on the microbial strain used and the specific mechanism of degradation; in-depth knowledge of metabolic activities for each microorganism is required for successful optimization of the degradation conditions. Cai et al. (2023) provided optimal conditions discovered for both fungal and bacterial PE degradation. The breakdown of various plastics, including PE, by *Pseudomonas* and *Arthrobacter* is optimized at temperatures between 30 °C–70 °C (Cai et al., 2023). Nutrient availability for the prosperity of microbial life is also important. Nitrogen and phosphorus are essential elements for the growth of microbes. A carbon-to-nitrogen ratio of 30:1 was described as optimal for microorganisms utilizing lignocellulosic degradation (Xie et al., 2022). The optimal ratio of phosphorus, in the form of $PO_{4}^{3-}$ , and potassium is described as 1:12 by Sun et al. (2022).

### 4.4 Microbial consortia for enhanced degradation

Individual bacterial strains, owing to their constrained metabolic capabilities, may not be fully effective in degrading PE when used in isolation. A strategic solution to this challenge is to create a synthetic microbial consortium where each specialized microbe contributes a specific enzyme to the overall PE degradation pathway (Salinas et al., 2025). Research has shown that implementing microbial consortia rapidly enhances PE degradation. Salinas et al. (2024) reported an 18% weight loss of LDPE when exposed to C2 consortia (comprised of *B. subtilis* RBM2, *F. oxysporum* RHM1, and *A. alternata* RHM4) in just 30 days. This is compared to the degradation of LDPE by *P. aeruginosa* WD4, which is around 9% after 100 days. Evidence of the success of such consortia in overcoming single-strain degradation of PE was provided. Due to the absence of a standardized set of analytical criteria for confirming PE biodegradation, assessments of PE degradation via microbial consortia often rely on indicators such as PE deterioration and weight loss (Liu et al., 2025). Nonetheless, the use of microbial consortia for PE breakdown is advantageous over single bacteria as it decreases the required microbial contact time and maximizes PE degradation.

## 5 Upcycling PE-derived small molecules into value-added products

**FIGURE 3:**
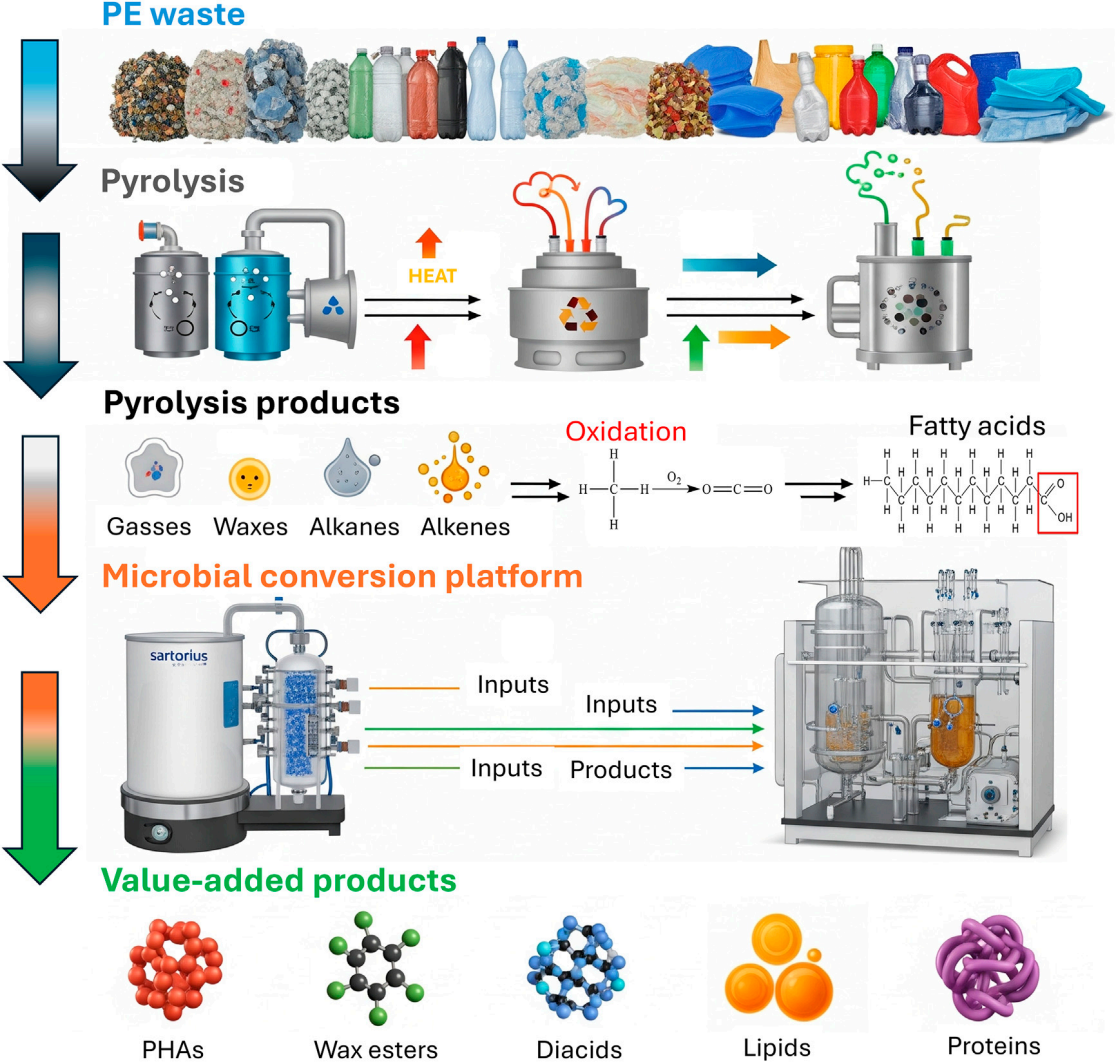
An integrated biorecycling and upcycling pipeline for PE waste.

Directly applying plastic-degrading microbes or enzymes to plastics in the environment presents significant logistical challenges. However, a viable approach involves using enzymes to first break down plastic waste into its constituent monomers or using pyrolysis to convert plastic waste into smaller products. These simpler products can then be utilized as a carbon source (feedstock) for microbial growth, allowing microorganisms to undergo bioconversion into value-added chemicals under controlled conditions (Xu et al., 2023; Suresh et al., 2025). This process is often referred to as biocatalytic upcycling, providing a promising strategy for managing plastic waste. An integrated multistage upcycling pipeline for PE waste is illustrated in Figure 3, where PE waste is collected and segregated from disposal lands and water bodies, and converted into aliphatic hydrocarbons like alkanes and alkenes, pyrolysis oil, and gas products via a pyrolysis process, followed by their utilization as a substrate for microbial conversion platforms to produce a variety of value-added products, including waxes, lipids, diacids, proteins, and polyhydroxyalkanoates (PHAs) for various industrial applications such as lubricants, cosmetics, biofuels, nylon monomers, food or feed ingredients, and biodegradable plastics.

### 5.1 Strategy and pathways for converting decomposed PE into value-added products

Thermochemical routes of PE upcycling for the formation of molecules such as C_1_-C_40_ mono-olefins, alkanes, and alkenes suffer from low productivity, low selectivity, and low product value (Zhao et al., 2020). Biological recycling alone is not a viable solution, as PE is extremely resistant and very stable. Researchers showed slow degradation rates of PE as the major challenge in this area of research (Abd El-Rehim et al., 2004). Only 1-3 wt.% loss was reported in 40–60 days of PE degradation using microbial systems (Ghatge et al., 2020). Furthermore, the current focus is more inclined towards the growth of microbial cells, with CO_2_ as a major product, instead of PE degradation. Therefore, a novel PE degradation approach is needed to address the PE waste problem. A hybrid approach for PE upcycling consists of PE oxidative pyrolysis as a first stage, by utilizing porous catalysts with appropriate active redox metal oxide sites, for decomposing waste PE into alcohols, aldehydes, C_5_-C_30_ alkanes, and carboxylic acids as a major PE decomposition product (Yeo et al., 2024). The following step consists of utilizing engineered microbial strains for the conversion of PE decomposition products and their intermediates into a series of value-added chemicals (Yeo et al., 2024).

**FIGURE 4:**
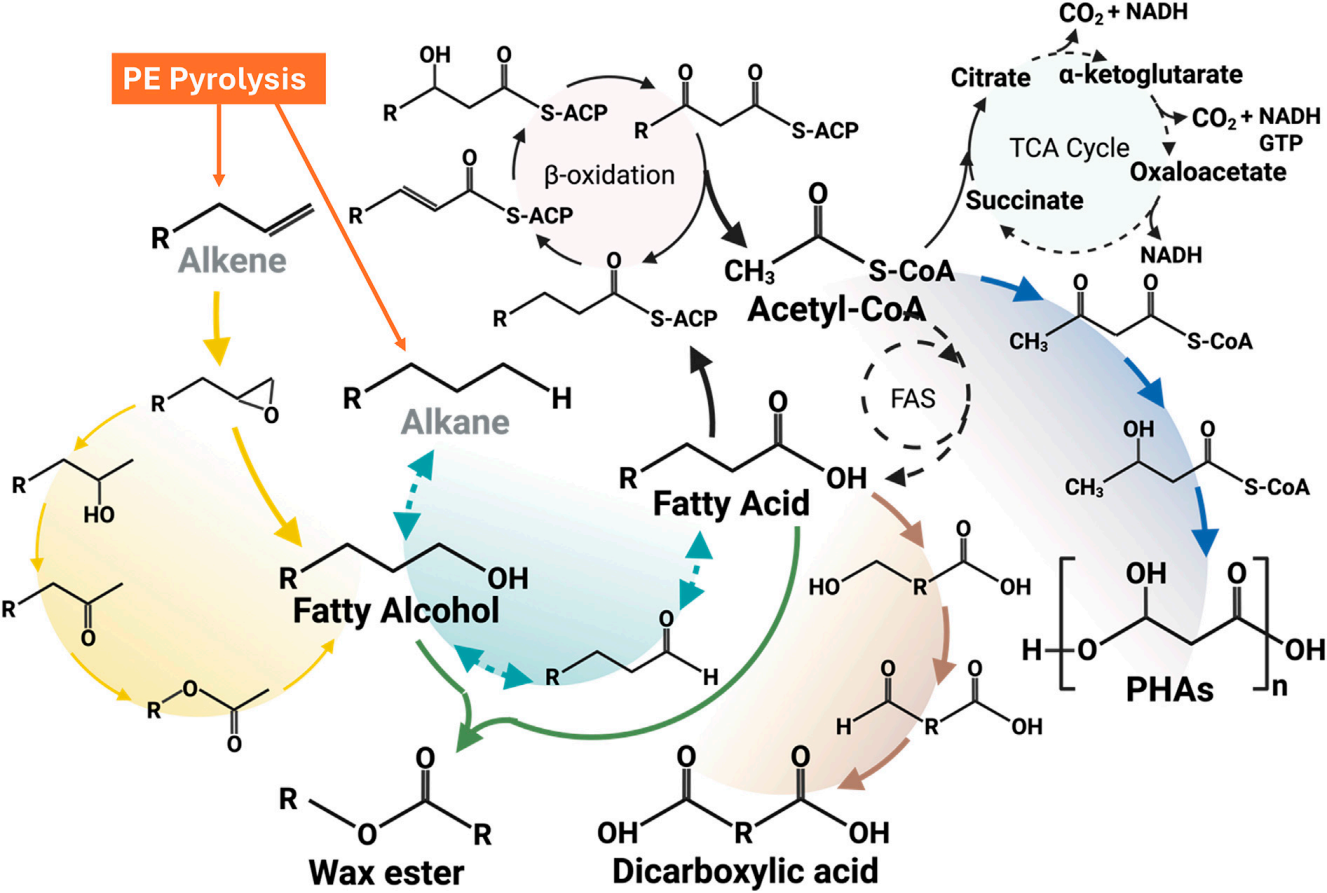
Major metabolic pathways for converting PE-derived alkanes and alkenes into value-added products.

Zhao et al. carried out PE pyrolysis at 500◦C–550 °C and showed the production of some aromatics (<5%), alkanes (10% or less), alkadienes and cycloalkanes (20%–30%), and mostly liquid products (50%–60%) (Zhao et al., 2020). Some potential microbial platforms (like yeast) can potentially utilize some of the PE decomposition products (like alkanes) to produce value-added chemicals (like dicarboxylic acids) by converting the alkane’s terminal methyl group and fatty acids via ω-oxidation into the carboxylic groups, followed by metabolizing fatty acids and dicarboxylic acids by undergoing a β-oxidation pathway (Lee et al., 2018), as shown in Figure 4. Thermal oxo-degradation (TOD) at 500 °C in an oxidative, noncatalytic environment breaks down HDPE into a mix of hydrocarbons, alcohols, aldehydes, and carboxylic acids suitable for microbial use (Brown et al., 2023). *Candida maltose*, *Scheffersomyces stipites*, and *Saccharomyces cerevisiae* were tested for their ability to grow on TOD products as the sole carbon source. The enriched consortia primarily converted the model alkane hexadecane into a C_16_–C_16_ wax ester, as reported by Gregory et al. (Gregory et al., 2022). This highlights the potential for using such microbial systems to produce valuable wax esters following the metabolic pathways as shown in Figure 4. PHAs are valuable biopolymers with wide-ranging industrial applications, and they can be produced through several metabolic pathways, including acetoacetyl-CoA synthesis, fatty acid biosynthesis, and fatty acid β-oxidation. The choice of pathway depends on the bacterial strain and the substrates provided (Vicente et al., 2023). The preferred metabolic pathway to produce PHAs using PE-derived substrates is shown in Figure 4. Regardless of the pathway, all routes converge at the same final step, which is catalyzed by the key enzyme PHA synthase (PhaC). This highlights the versatility of microbial systems in producing a variety of value-added products. Furthermore, genetic engineering offers powerful tools to enhance and modify these metabolic pathways, enabling the biosynthesis of novel and valuable biochemicals. Through targeted genetic modifications, microbes can be engineered not only to improve yield and properties but also to upcycle PE degradation products into new value-added products (Connor et al., 2023). Table 2 summarizes the variety of value-added products reported in the literature by utilizing PE-derived feedstocks as a carbon source.

**TABLE 2:** Microbial platforms and products from polyethylene (PE)-derived feedstocks.

Value-added product	Microbial strain	PE-derived feedstock	Biomass growth	Production titer/rate/yield	Ref.
Silk protein	*Pseudomonas aeruginosa *RR1	Hexadecane (C_16_)	>1 × 10^9^ cfu/mL	11.3 ± 1.1 mg/L	Connor et al. (2023)
Lipid	*Yarrowia lipolytica* DSM1345	PE pyrolysis oil (C_9_-C_16_)	OD600 3.56	122 mg/L	Zhou et al. (2023)
Polyhydroxyalkanoate (PHA)	*Pseudomonas putida* KT2440	PE-derived fatty acid mixture	83.0 g L^-1^ CDW	65 (% CDW)	Guzik et al. (2021)
Wax esters (Cetyl palmitate)	*Rhodococcus aetherivorans* consortia 1 (E1)	Hexadecane (C_16_)	Not discussed	35.6 mg/g CDW	Gregory et al. (2022)
	*Rhodococcus aetherivorans* consortia 2 (E2)		Not discussed	30.18 mg/g CDW	
Wax esters (Myristyl palmitate)	*Rhodococcus aetherivorans* consortia 1 (E1)	Hexadecane (C_16_)	Not discussed	3.6 mg/g CDW	
	*Rhodococcus aetherivorans* consortia 2 (E2)		Not discussed	6.0 mg/g CDW	
Polyhydroxyalkanoate (PHA)	*Ralstonia eutropha* H16	Oxidized PE wax	3.66 g L^-1^ CDW	1.24 g L^-1^	Radecka et al. (2016)
Medium-chain α,ω-diacids (C_7_–C_14_)	*Candida tropicalis* Ct6	Mixed plastic with PE, pyrolysis oil (C_10_-C_12_ alkanes)	OD600 ∼57	100% yield	Yeo et al. (2024)
Polyhydroxyalkanoate (PHA)	*Cupriavidus necator *H16 ATCC 17699	Non-Oxygenated PE Wax	1.42 g L^-1^ CDW	0.46 g L^-1^	Johnston et al. (2017)
Protein	Microbial consortia (*Spurr River sediment cultures*)	Linear alkenes (C_5_ to C_25_)	Not discussed	0.136 g	Byrne et al. (2022)
	Microbial consortia (*farm compost cultures*)		Not discussed	0.010 g	
Polyhydroxyalkanoate (PHA)	*Pseudomonas aeruginosa *PAO-1	PE pyrolysis wax	∼10 g L^−1^ CDW	25 (% CDW)	Guzik et al. (2014)
	*Pseudomonas aeruginosa *GL-1		∼0.39 g L^−1^ CDW	∼18.9 (% CDW)	

### 5.2 Major products from the bioconversion of PE pyrolysis-derived feedstocks

#### 5.2.1 Long-chain dicarboxylic acids

Alkanes-assimilating microorganisms, mainly yeast, are potential candidates for the biomanufacturing of dicarboxylic acids (DCA). *Candida tropicalis*, an alkane-assimilating yeast strain, shows a strong potential to convert PE-derived alkanes into DCA via ω-oxidization (Lee et al., 2018). Several efforts have been made in the past to increase the DCA yield either by enhancing the ω-oxidization pathway and/or by blocking the β-oxidation pathway to redirect the metabolism of fatty acids and alkanes towards the production of valuable DCA (Picataggio et al., 1992). The bioconversion efficiency has been further enhanced by overexpressing the cytochrome P450 monooxygenase and NADPH–cytochrome reductase genes, which encode the rate-limiting ω-hydroxylase enzyme involved in the ω-oxidation pathway of alkanes (Picataggio et al., 1992). Figure 4 shows the general metabolic pathway to produce dicarboxylic acids using alkanes and alkenes as feedstock. Currently, the majority of long-chain DCA (≥C_10_) production takes place in China, where pure alkanes are fermented using *Candida tropicalis* strains developed through traditional physical and chemical mutagenesis. These strains can produce over 130 g/L of dodecanedioic acid (DDDA) from decane (Xu CY, 2002). An interesting study by Yeo et al. (2024) demonstrated the potential of converting mixed plastic waste, including PE, into valuable DCAs using a chemo-biological approach. Pyrolysis oil, obtained from household plastic waste and rich in hydrocarbons (C_7_–C_32_), was processed using a genetically engineered *Candida tropicalis* strain with a blocked β-oxidation pathway to produce α,ω-diacids. Medium-chain hydrocarbons were extracted by distillation at 200 °C and hydrogenated to mitigate toxicity, enabling successful microbial conversion. Notably, the cells were able to sustain growth even in the presence of an 8% concentration of hydrogenated compounds. A successful biotransformation resulted in 94.3% of the α,ω-diacids in the range of medium-chain length (C_7_ to C_14_) (Yeo et al., 2024). Despite the growing interest in upcycling plastic waste, studies specifically focused on the synthesis of α,ω-DCAs from PE-derived products remain limited. Therefore, the promising step toward valorizing PE waste highlights the need for further research into efficient and scalable DCA production from PE-derived substrates.

#### 5.2.2 Wax esters

Wax esters are of great industrial importance, with the longer chain lengths (range from C_32_-C_36_) having the highest value (Domergue and Miklaszewska, 2022). PE upcycling into wax esters has been reported by combining chemical catalysis and bioconversion for the volatilization of PE deconstruction products. Gregory et al. (2022) conducted studies in which microbial consortia were fed a mixture of PE-derived *n*-alkanes (ranging from C_4_ to C_35_). The main metabolite generated from the model alkane hexadecane by enriched consortia was identified as a C_16_–C_16_ wax ester. The incubation of *Rhodococcus aetherivorans* consortia 1 (E1) and *Rhodococcus aetherivorans* consortia 2 (E2) with hexadecane led to the formation of hexadecanol within 24 h, followed by the production of hexadecanoic acid after 48 h, indicating the existence of terminal oxidation. Quantitative analysis after 14 days in nitrogen-limited medium revealed cetyl palmitate (C_16_–C_16_ ester) as the major metabolite for both E1 (35.6 mg/g CDW) and E2 (30.18 mg/g CDW), as reported in Table 2. Additionally, along with other value-added compounds such as hexadecanoic acid, lauryl palmitate (C_16_–C_12_ ester), and myristyl palmitate (C_16_–C_14_ ester), 1-hexadecanol was identified as the second most abundant metabolite (Gregory et al., 2022). Bacterial mechanisms have been studied for utilizing such hydrophobic substrates, describing the enzymatic pathways involved in their degradation and transformation (Wentzel et al., 2007). However, very few studies explored the PE upcycling into wax esters, highlighting a promising area for future research, due to the anticipated growing demands for biological waxes, especially wax esters, in the pharmaceutical, food, and lubricant industries (Wentzel et al., 2007).

#### 5.2.3 Polyhydroxyalkanoates (PHAs)

Upcycling of PE using microbial systems has been extensively studied for the production of PHAs as a value-added biodegradable polymer (Sohn et al., 2020). The PE is first pyrolyzed to generate hydrocarbon wax, which is then oxidized to produce a mixture of fatty acids. After purification, this mixture served as the carbon source for microbial growth and selection (Guzik et al., 2021). The study carried out by Guzik et al. (2021) focused on optimizing the production of medium-chain-length polyhydroxyalkanoate (mcl-PHA) using *Pseudomonas putida* KT2440, fed with a fatty acid mixture derived from PE by a chemo-biotechnological approach. Following initial screening in shake flasks, *Pseudomonas putida* KT2440 was selected for scale-up studies in bioreactor experiments. *P. putida* KT2440 was fed with PE-derived fatty acids in a 20-L bioreactor. The fermentation process achieved high yields of 83.0 g/L cell dry weight, with 65% of that being PHA, within just 25 h. The initial exploration of PE upcycling in PHA was carried out by utilizing PE pyrolysis wax, which was produced by breaking down low molecular weight PE into PHA. Later, transition-metal-catalyzed oxidation resulted in the formation of a fatty acid mixture instead of paraffin waxes, which enhanced the biomanufacturing efficiency of PHA owing to better solubility (Schwab et al., 2024). Another study carried out by Ekere (Ekere et al., 2022) reported a novel recycling method for waste Tetra Pak^®^ packaging PE materials. The polyethylene-Tetra Pak (PE-T) component of this packaging material, obtained via a separation process using a “solvent method”, was utilized as a carbon source for the biosynthesis of PHAs by the bacterial strain *Cupriavidus necator* H16. Bacteria growth after 48–72 h, at 30 °C, in TSB (nitrogen-rich) or BSM (nitrogen-limited) media supplemented with PE-T resulted in the accumulation of 40% w/w PHA in TSB fed with PE-T. 1.5% w/w PHA in the TSB control, and no PHA was detected in the BSM control. It is well known that the PHAs are usually synthesized by microbes within their cells and stored as carbon and energy reserves in specialized sub-cellular structures called carbonosomes, particularly under nutrient-limited conditions (Tanamool et al., 2013). Figure 4 also illustrates the metabolic pathway for converting PE pyrolyzed products into PHAs. Several studies carried out to produce PHAs using PE-derived feedstocks are summarized in Table 2.

#### 5.2.4 Recombinant proteins

*Pseudomonas* bacteria are especially notable for their ability to efficiently use both individual alkanes and mixtures of alkanes as the sole carbon source to support their growth (Chayabutra and Ju, 2000; Obayori et al., 2009). PE-derived substrate was reported to be successfully converted into recombinant proteins by utilizing a novel *Pseudomonas* bacterial strain using a two-step process (Connor et al., 2023). Connor et al. (2023) reported a biomass development of up to 1 × 10^9^ cfu/mL by utilizing hexadecane from PE decomposition product as a sole carbon source. The chemically depolymerized PE, containing a mix of branched and unbranched alkanes, was reported to be converted by *Pseudomonas aeruginosa* into silk protein, achieving titers of 11.3 ± 1.1 mg/L. Byrne et al. (2022) utilized HDPE undergone a pyrolysis process, which resulted in a mixture of C_5_-C_25_ alkene compounds that served as the primary substrate. These compounds were fed to enrich cultures from six different environmental inocula, including vermicompost, mud, and river sediments. The microbial communities in these cultures assimilated the alkene compounds, converting them into cellular biomass and thus producing proteins. After 5 days, the cultures showed an increase in protein content ranging from 0.010 g to 0.136 g, depending on the inoculum source. This shows the potential of producing recombinant proteins using PE-derived products as a feedstock. However, very limited work has been done in this area, and considerable future potential is evident.

## 6 Future perspectives and challenges

Biorecycling and upcycling are capable solutions for their replacement with the less efficient, disproportionately high-cost, and/or ecologically harmful conventional methods, including landfill, mechanical, and chemical recycling, to overcome the PE waste problems. Although all recycling strategies have their associated carbon footprints, mechanical and chemical recycling, compared to biological recycling, are constrained by product downcycling, quality deterioration, and limited recycling cycles, ultimately failing to achieve a fully circular carbon system. Mechanical recycling avoids about 25 wt.% of CO_2_ emissions due to the low quality of recyclates (Vollmer et al., 2020). The results indicate that mechanical recycling of LDPE has a significant impact on global warming, corresponding to 152 kg CO_2_-eq per 1,000 kg of LDPE (Ruggeri et al., 2025). In contrast, another study reported an emission burden of 324.64 kgCO_2_-eq per 1,000 kg using anaerobic digestion treatment (Gadaleta et al., 2022). However, pyrolysis, as a part of a two-stage upcycling strategy, can substitute fossil-fuel-based feedstocks, avoiding roughly 30 wt.% of incineration-related CO_2_ emissions (Vollmer et al., 2020). Biorecycling and biological upcycling, while still in early stages, offer transformative potential by converting PE waste into value-added chemicals. With continued advancements in enzyme engineering, process integration, recirculation of carbon from byproducts as a feedstock, and renewable energy use, the carbon intensity of biorecycling and upcycling is expected to decrease, thereby questioning the assumed superiority of conventional recycling approaches. PE biorecycling can be catalyzed using purified enzymes and enzyme cocktails for better performance. Bacterial and fungal degraders are also potential candidates for their implementation in future scale-up studies of PE degradation. However, cell-free systems show potential advantages over bacterial and fungal systems in terms of mild reaction conditions and applications in areas where conditions are unsuitable for fungal and bacterial cultures. The key challenge in the industrial applicability of PE biorecycling is the nature of mixed PE waste, containing tons of different types of PE single-use products, possessing a range of thermostability, crystallinity, and structural properties. Use of enzyme cocktails can be a potential solution to overcome a mixed PE waste problem, since enzyme cocktails are already implemented in industries for complex compounds degradation, such as lignocellulose (Lopes et al., 2018). Combining diverse proteins and functional domains creates new possibilities for developing innovative PEases. For instance, the use of surface adhesion proteins along with PEases could potentially increase the overall PE degradation (Zhang et al., 2023). Utilizing microbial consortia is another promising method for industrial biorecycling of PE (Skariyachan et al., 2021). Even though enzyme cocktails and microbial consortia show candidacy, there are several major challenges to their industrial applicability. Interpreting currently available data on the biological breakdown and metabolism of PE is challenging because many studies depend on relatively imprecise and criticizable analytical techniques, including weight loss measurements, microscopy, and infrared spectroscopy, to demonstrate PE degradation (Stepnov et al., 2024). Further complications in the widespread applicability of such technologies lie in the degradation demonstration of PE that is not pure (e.g., containing metabolizable additives) (Cuthbertson et al., 2024), resulting in the overestimation of PE recycling efficiency. Furthermore, very limited data is available for industrial-scale economic modeling and feasibility analysis of PE biorecycling methods (Verschoor et al., 2022). However, if PE can be utilized as the sole carbon source for microbes or microbial consortia at an industrial scale, biorecycling and upcycling of PE waste would become a highly effective solution (Verschoor et al., 2022; Bergeson et al., 2024).

Utilizing PE waste as a potent feedstock for microbial systems for biorecycling and its upcycling into value-added chemicals also poses bottlenecks and challenges in terms of bioreactor design, continuous processing optimization, and feedstock logistics (Bergeson et al., 2024). PE-derived monomers, as well as partially degraded PE, serve as critical feedstocks within the upcycling framework for designing new processes to produce value-added chemicals and biofuels (Hou et al., 2021). In addition to the uncertain characteristics of the PE-derived feedstock obtained from processes like pyrolysis, the large-scale industrial implementation of waste PE pyrolysis faces several operational hurdles (Orozco et al., 2022). These challenges primarily stem from the low thermal conductivity and adhesive properties of PE, along with the energy-intensive (endothermic) nature of the pyrolysis process. As a result, designing an appropriate pyrolysis reactor with a suitable catalyst is essential to ensure efficient and controlled plastic conversion for its efficient upcycling (Solis and Silveira, 2020; Orozco et al., 2022). Several microbial strains, as listed in Table 2, can utilize PE-derived feedstocks for their conversion into value-added products. However, until now, PE upcycling using microbial strains has largely been limited to the production of PHAs as a value-added product (Sohn et al., 2020; Connor et al., 2023). Very few studies focused on the production of proteins, lipids, and diacids, as provided in Table 2. Thus, there are a lot of future opportunities to broaden the variety of value-added products obtainable through microbial upcycling of PE. For such a purpose, it is necessary to employ novel microbial and genetically engineered strains with efforts in genetic manipulation, genetic modification, recombinant DNA technology, and gene cloning to engineer metabolic pathways for the cutting-edge biomanufacturing of new products via upcycling of PE waste (Rezaei et al., 2024).

The integration of omics technologies like proteomics, genomics, metabolomics, and transcriptomics has transformed the future of microbiome research for plastic waste biorecycling by providing deeper knowledge into microbial systems (Yang et al., 2025). Multi-omics technologies are quite significant in gut microbiome research, where microbial distribution patterns influence host interaction and functional roles (Tropini et al., 2017). The application of machine learning and statistical models to identify non-intuitive patterns between input features and experimental outcomes in metabolic engineering research has been limited. However, there is potential to utilize machine learning in active learning frameworks for accelerated development of biochemical production strains for high-yield biomanufacturing of value-added chemicals (Kumar et al., 2021). Recently, a new PEase was discovered from *Lysinibacillus fusiformis* via a combination of computational structure analysis and preliminary activity-based screening (Zhang et al., 2023). Despite recent advancements, the utilization of omics technologies in PE biodegradation remains limited. In the case of microbial degraders, only about 2% of environmental microorganisms can be cultured under laboratory conditions, leaving many unexplored fungi, extremophiles, and several bacteria, presenting substantial opportunities for future research (Wade, 2002). Future design approaches are likely to focus on customizing enzymes for targeted properties. Deep learning determined *de novo* enzyme design that offers promising prospects for creating highly efficient PEases (Jin et al., 2023). Furthermore, directed evolution plays a crucial role in enhancing enzyme selectivity, stability, surface adhesion, and degradation efficiency of PEase.

Although active research is underway in the field of PE biorecycling and its upcycling into value-added chemicals, a supportive policy and regulatory framework is essential to drive the adoption and expansion of these solutions. This includes setting clear regulatory frameworks, promoting collaboration between industry, academia, and government, and introducing financial incentives to encourage the development and placement of biorecycling technologies. Establishing a regulatory framework that outlines quantities and types of PE allowed, along with required pre-treatment procedures and monitoring protocols, is also crucial. Engaging local communities and stakeholders in decision-making can help address PE waste concerns collaboratively. It is also important to highlight that Life Cycle Assessment (LCA) is utilized to explore new biorecycling approaches. For instance, Wang et al. (2021) examined the environmental impacts of combining biorecycling via anaerobic digestion with pyrolysis. Integrated pathways (biorecycling and pyrolysis) to standalone processes were compared. Their findings showed that the combination of anaerobic digestion with pyrolysis notably reduces the overall environmental impact, identifying it as the most sustainable option among those assessed. Hernández et al. (2023) carried out a detailed techno-economic analysis and LCA study of PE waste upcycling into several value-added products. Their study demonstrated pyrolysis of PE followed by conversion to lubricant oils as the most economically favorable technology. LCA of PE pyrolysis, involved in the upcycling of PE, indicates that the primary environmental advantages come from substituting fossil-derived products with the oils and chemicals (alkanes, alkenes, waxes, and gases) produced through this process (Garcia-Garcia et al., 2024). Along with LCA, carbon neutrality analysis is also crucial to evaluate the environmental performance of the proposed biorecycling and upcycling technologies (Zhang et al., 2024). Implementation of integrated and non-conventional technologies is yet to be studied for the range of PE waste streams. Continued future progress is expected to contribute significantly to overcoming PE waste accumulation through effective biorecycling and upcycling strategies.

## 7 Conclusion

The growing accumulation of PE waste poses a significant environmental challenge, driving the need for sustainable recycling and upcycling strategies. This review highlights up-to-date findings and discoveries for PE biorecycling using both enzymatic and microbial systems. PE pretreatment provides a potential for better decomposition performance. Microbial consortia and the fusion of enzymes would be a better option for higher-scale PE decomposition compared to using a single microbe or enzyme. However, the area of PE biorecycling using enzymes and microbes still needs more exploration and better understanding to discover or identify specific enzymes responsible for PE decomposition and decomposition pathways, along with the exploration of combining enzymes, strains, and process engineering. A hybrid chemical-biochemical conversion approach for upcycling PE into value-added products shows great potential, but current research data is very limited to come up with an industrially applicable approach. The first stage of an upcycling technique has its challenges in terms of lower PE decomposition product yield to effectively utilize decomposition products as a feedstock for the second stage of biomanufacturing using microbial strains. Initial PE decomposition includes oxidative and catalytic treatments, breaking down PE into soluble intermediates such as paraffins and fatty acids, which enhance microbial uptake and bioconversion efficiency. Very few PE upcycling products have been explored till now. Acquired product titers are low, and a limited understanding of the metabolic pathways makes it challenging to improve product titers. Future metabolic engineering with the assistance of computer-aided and omics technologies is required to make biomanufacturing of value-added products from waste PE an environmentally benign and economically competitive option.
